# Usability evaluation of emergency information systems in educational hospitals in Kerman, Iran

**DOI:** 10.1186/s12911-023-02357-3

**Published:** 2023-11-30

**Authors:** Farzaneh Behnam, Reza khajouei, Amir Hossein Nabizadeh, Saeed Saedi, Mohammad Mahdi Ghaemi

**Affiliations:** 1 Medical Informatics Research Center, Institute for Futures Studies in Health, Kerman University of Medical Sciences, Kerman, Iran; 2 Department of Health Information Sciences, Faculty of Management and Medical Information Sciences, Kerman University of Medical Sciences, Haft-Bagh Highway, PO Box 7616911313, Kerman, Iran; 3 Medical Informatics Research Center, Institute for Futures Studies in Health, Kerman University of Medical Sciences, Kerman, Iran/INESC-ID, Lisbon, Portugal; 4 Shirvan Center of Higher Health Education, Imam Khomeini Hospital, North Khorasan University of Medical Sciences, Bojnurd, Iran

**Keywords:** Emergency information system, Hospital information system, Usability, Evaluation

## Abstract

**Background:**

Smart and practical health information systems and applications with fewer errors are crucial for healthcare facilities. One method that ensures the proper design of health information systems (HIS) and applications is usability evaluation.

**Objective:**

This study aimed to evaluate the usability of the emergency information systems used at the emergency departments of four educational hospitals in Kerman, Iran.

**Method:**

This study was conducted in two phases. In the first phase, the information systems' errors and shortages were identified using a semi-structured questionnaire by users (nurses and the IT staff). In the second phase, based on the results of the first phase, two questionnaires were designed for each group of users to their opinions about the usability of the emergency information systems.

**Results:**

The average score of “reducing and facilitating user’s daily activities” was significantly different among hospitals (*p* = 0.03). Shahid Beheshti Hospital obtained the lowest usability score (17.5), and Afzalipour Hospital received the highest usability score (21.75). Moreover, the average score in “use of the HIS” for nurses and IT staff was 2.93 and 3.54 on a scale of 5, respectively.

**Conclusion:**

Usability evaluation of health information systems is essential to ensure that these systems provide sufficient and accurate information and requirements for users and health care providers. Also, modifying health information systems based on the user views and expectations improves the quality of the system and user-system Interaction.

## Introduction

Promoting community health is possible by providing quality healthcare services. Healthcare providers need tools and strategies to improve their services [1, 2]. Recent advances in information technology and computer science have led to the establishment and development of information systems in various fields. Studies in management information systems have shown that using computer-based applications can positively affect the performance of different organizations, such as healthcare organizations [3]. In healthcare, these systems are known as health information systems (HISs) and are used to facilitate management and clinical tasks [4]. In the healthcare organization, These systems positively affect both patients and staff. Furthermore, they enable us to monitor and access essential data for making clinical decisions, setting goals and following them through, and improving healthcare quality, patient safety, and system effectiveness. They also support health-related interventions [5–8].

Emergency Department Information System (EDIS) is a component of a hospital information system that plays a crucial role in information and care management as well as emergency department management processes [9, 10]. The emergency department is characterized by several significant features, including unpredictable admissions and the physical condition of patients, and its staff is under pressure to perform diagnostic-therapeutic procedures upon arrival and immediately [11–13].

The immediacy of data requirements, the often chaotic nature of the environment, and the volume and intensity of change make the emergency department (ED) one of the most challenging areas to manage in a hospital. These challenges, in turn, lead to irreparable consequences such as increased patient mortality, high costs, increased waiting time and patient dissatisfaction, increased medical errors, and the occurrence of violence and disruption in medical services [14–16].

Therefore, compared to other departments, the emergency department needs significant flexibility and immediate planning of resources [17]. Integrating the Emergency Room Information System "EDIS," specific workflows can be automated to reduce the risk of human error and eliminate supply chain and patient delays. They Effectively accelerate turnover while lowering costs, maintaining quality patient care [18], improving the performance of the emergency department [19], providing more accessible and faster access to patient information [20], recording much better and more accurate clinical and management information, helping to make decisions [20], and eliminating the limitations of paper systems Such as the simultaneous access of several users to information and the illegibility of information [21]. But despite its increasing use in recent years in different countries [22], EDIS has yet to be widely accepted and used and has yet to be evaluated [18, 23, 24]. Therefore, intervention to improve the performance of EDIS will have the most significant benefit for the treatment system.

Due to the critical importance of emergency operations, emergency information systems must be free of usability problems to avoid errors. In doing so, systems and applications must be appropriately designed and used by scientific principles [25]. One of the ways to ensure the proper design of health information systems and programs is to evaluate their usability. Evaluation also plays an essential role in software development [26, 27]. According to various studies, it is necessary to observe the principles of usability in the design of the EDIS user interface [28, 29]. The usability index evaluates the performance of a product in terms of user satisfaction and increased productivity [30–32]. Usability issues are the most frequent challenges that might discourage a user from using the information system [33].

When quality-determining parameters are used in a system, and the system is designed and implemented correctly, its usability can be ensured [34, 35]. The usability of a system reflects on the degree of its efficiency, effectiveness, and user satisfaction in reaching predetermined goals. It depends on user interactions, products, tasks, and the environment [36].

If a system has usability issues, its efficiency and effectiveness will be reduced. As a result, it will discourage the user from using it since it is likely that the user will lose trust in the reliability and usability of the system [37]. Various studies have been conducted over recent years to evaluate the usability of HISs and how to improve the procedures [38–40], often reporting high usability problems. These problems adversely affect how users interact with their systems [25, 41, 42], and Many of these studies have been conducted extensively in private hospitals, physicians' offices, or private healthcare institutions.

However, the Emergency Department Information System (EDIS) used in the educational hospitals of Kerman city, whose information systems Shafa Hospital, Afzalipour Hospital, and Beheshti Hospital have been designed by Tirajeh and Bahonar Hospital Peyvand Dadeha companies, have not yet been evaluated. Therefore, this study aims to assess the usability of the information systems in EDIS of these educational hospitals in Kerman. Suppose the information system of a healthcare organization has usability defects. In that case, it can disrupt the treatment processes that are carried out there, which would ultimately reduce the quantity and quality of offered services. Therefore, identifying possible defects in the system, if any, and resolving them will help improve the design, increase user satisfaction, and reduce hospital costs.

## Method

This was a cross-sectional study. This study was carried out at four public university hospitals in Kerman. The first hospital, Afzalipour Hospital, with emergency poisoning and suicide, obstetrics, and gynecology, children and internal medicine, 800 beds affiliated to Kerman University of Medical Sciences with HIS related to Tirajeh Company, the second hospital, Shafa Hospital, a public university hospital with emergency Cardiology, Nephrology, Neurology and Ophthalmology, 380 beds affiliated to Kerman University of Medical Sciences with HIS related to Circulation Company and the third hospital, Beheshti Psychiatric Hospital with 240 beds associated with Kerman University of Medical Sciences with HIS pertaining to circulation and fourth hospital, Bahonar University Hospital is a general university hospital with 530-bed injuries, orthopedics, and neurosurgery affiliated to Kerman University of Medical Sciences with HIS affiliated to the company.

The purpose of this study was to evaluate the usability of the information system of the ES from the users’ (i.e., nurses and the IT staff) perspectives to identify possible issues and compare them in different hospitals. For that, the study was conducted as follows:

- *First phase: identifying the defects and shortcomings of the HIS through interviews*
  The aim here was to collect and evaluate the users' opinions about the HIS of the Emergency Section of their hospital to identify any possible defects and usability issues in the system. A semi-structured face-to-face interview was conducted with all personnel working in the hospital’s Emergency Section (supervisors, head nurses, nurses) and IT staff working in the Computer Section. The interviewer explained to all participants that the purpose of the study was to identify the possible issues and shortcomings of the information systems that the staff may have experienced when working with them. Interviews continued until data saturation was obtained. Studies have shown that experimental data are saturated with 9–17 discussions [43, 44]. In all interviews, the participants’ responses were recorded immediately. In the next phase, a questionnaire was designed to evaluate the usability of these information systems.

- *Second phase: evaluating the usability of HIS in Emergency Sections*
  Here, the aim was to evaluate the usability of the HISs in the Emergency Sections from the users' perspectives and to compare the two employed HISs in ES developed by Tirajeh and Peyvand Dadeha. The study population included nurses, as users of unique terms, and the IT staff at the Computer Section, as the system’s supporters at the educational hospitals affiliated with Kerman University of Medical Sciences.

For this purpose, based on the results obtained from the first phase, two separate questionnaires were designed to evaluate the usability of the information systems from the perspectives of the two groups of users. The validity of the questionnaires was confirmed by two medical informatics specialists and a senior medical informatics expert using the validity method. The reliability of the questionnaires was confirmed using Cronbach's Alpha test (α = 0.82). A group of participants answered each questionnaire. Before distributing the questionnaires, participants were informed about the purpose of the study and their consent for participation was obtained. They were assured that their information would remain confidential.

A questionnaire that was answered by the IT staff contained 37 questions to study their experience in using the information systems. These questions focused mainly on four categories: the system’s security (19 questions), the system’s compliance with standard criteria (9 questions), the system’s usability (6 questions), and the system’s connectedness (3 questions). The other questionnaire that was designed for the nurses held 50 questions in 8 categories: reduction/facilitation of user's daily activities (11 questions), removal of the costs of the organization (2 questions), an increase of the accuracy and quality of services (4 questions), system’s security (11 questions), system’s ease of use (8 questions), data quality (5 questions), system’s stability (4 questions), and user’s satisfaction (5 questions). Both questionnaires were based on a 5-point Likert scale where the participants had to select just one answer for each question (ranging from significantly less, less, to some extent, much, very much). SPSS software version 24 was used for data analysis.

## Result

### First phase

From the interviews, the issues that users had with the HIS system of the studied hospitals were identified (Table 1).

**Table 1:** the identity of the users’ issues with the HIS system of the four hospitals

Issues at the Triage	Issues at CPR	Issues at Acute 1 and 2
Entering and recording data manually in the triage sheet, the triage registry book, and the HIS system of the hospital is massive time consumption	Nurses and residents prefer to record diagnostic information and write reports manually	When patients are transferred from one section to another, their information is not automatically and wholly transferred to the new section and needs to be moved manually
As the computer system crashes constantly and the IT staff is not responsive to solving technical issues, the staff prefers to record the information on paper and manually	Different devices used in CPR, like monitoring devices(which display the patient’s ECG, blood oxygen, blood pressure, and heart rate), electroshock, suction, etc., are not connected to the HIS system in which their information should be recorded	Radiology requests are sent on paper, and we do not have an electronic connection with the RIS system
All users use a shared account( each user is not provided with a single account)	The system fails to warn that a patient with a stable condition has exceeded their length of hospitalization(significantly if it has surpassed 6 h) to inform the physician to release them	
Failure to recognize the real identity of the user in case of forgetting the previous user to log out of the system	Nurses’ reports are paper-based	
An independent search module has yet to be defined to search for patients and enter their information. The user must refer to the new patient’s registration module, enter their data, and start searching		
Lack of standard coding in the system for diseases and patients’ problems		
Lack of a responsive decision-support system that would eliminate over-triage and under-triage errors when the nurse has determined a patient’s state		

### Second phase

Evaluating the usability of the Information System of the Emergency Section.

A total of 101 questionnaires were collected; the nurses completed 85 and 16 by the IT staff. The average age of the nurses was 32.28 years; 86% of them were women, and most had a bachelor’s degree. The average age of staff was 35.56 years. Similarly, 68% of them were women, and most had a bachelor’s degree (Table 2).

**Table 2:** Description table of demographic data

	nurses				Computer staff			
	minimum	maximum	average	Standard deviation	minimum	maximum	Average	Standard deviation
age	22	60	32.28	7.668	27	41	35.56	4.633
Gender^a^	0	1	-	-	0	1	-	-
Education^b^	1	3	-	-	2	3	-	-
Hospita^c^	1	4	-	-	1	4	-	-

^a^Gender: woman = 0, men = 1

^b^Education: 1: Associate Degree, 2: Bachelor’s Degree, 3 Master’s Degree

^c^Hospitals: 1: Afzalipour, 2: Bahonar, C: Shafa, D: Shahid Beheshti

Data analysis showed that the participants’ gender had no significant effect on their responses (*p* >  = 0.320) (Table 3).

**Table 3:** The effect of the participant’s gender on the participant’s response

	The average score of men	The average score of women	*P*-Value
Nurses’ questionnaire			
Reducing or facilitating user’s daily activities	32.35	32.72	0.495
Reducing the costs of the organization	6.24	6.26	0.776
Increasing the quality and accuracy of offered services	11.94	12.07	0.517
System’s security	31.50	31.85	0.582
System’s ease of use	23.56	23.75	0.84
Quality of data	14.55	14.73	0.854
System’s stability	11.66	11.80	0.827
User’s satisfaction	14.79	15.04	0.463
IT staff’s questionnaire			
System’s security	68.4	67.54	1
System’s compliance with standard criteria	32.80	34.73	0.583
System’s usability	20.6	20.27	0.661
System’s connectedness	10.20	8.64	0.32

In all groups, the participants’ age had no significant effect on their responses to the usability questions (*p* >  = 0.111). Data analysis also showed that the participants’ level of education, in both women and men, had no significant effect on their responses (*P* >  = 0.086) (Table 4).

**Table 4:** The effect of the participant’s level of education and the participant’s response

	Associate Degree	BSc	MSc	*P*-Value
Nurses’ questionnaire				
Reducing or facilitating user’s daily activities	26.05	32.94	31.8	0.27
Reducing the costs of the organization	5	6.23	7.2	0.105
Increasing the accuracy and quality of offered services	11.5	12.06	12.4	0.9
System’s security	30	31.9	31.8	0.645
System’s ease of use	20	23.73	25.6	0.435
Quality of data	14.5	14.65	16	0.943
System’s stability	11.5	11.78	12.2	0.942
User’s satisfaction	11	15.04	17	0.2
IT staff’s questionnaire				
System’s security	-	68.5	66.67	0.913
system’s compliance with standard criteria	-	33.7	34.83	0.549
System’s usability	-	31.1	19.17	0.086
System’s connectedness	-	9.5	8.5	0.401

### The nurses

**Fig. 1:**
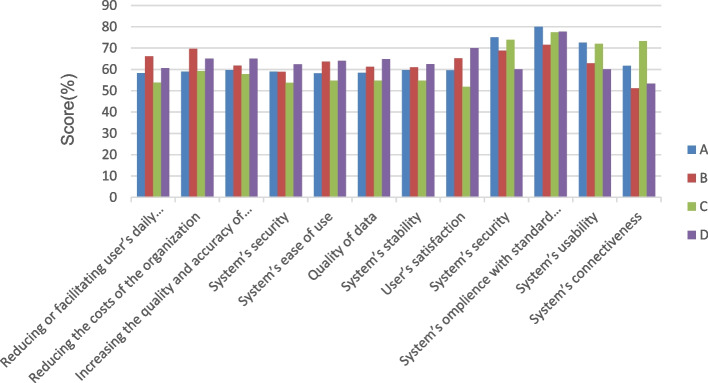
A demonstration of the average of all the different usability components in each hospital A: Afzalipour Hospital, B: Bahonar Hospital, C: Shafa Hospital, D: Shahid Beheshti Hospital

The average usability scores of all four hospitals (HIS) are presented in Fig. 1. There was a significant difference among the hospitals investigating how their information systems have helped reduce/ facilitate the users’ daily activities (*p* = 0.03). Comparing the nurses’ perspectives about the HIS of their hospitals in pairs showed a reduction/facilitation of users’ daily activities and a reduction of costs between Afzalipour Hospital and Bahonar Hospital (*p* <  = 0.016). When comparing Afzalipour Hospital and Shahid Beheshti Hospital, a significant difference was observed in their data quality (*p* <  = 0.037).

In comparing Afzalipur and Shafa hospitals, all groups had no statistically significant differences (*P* >  = 0.112). In comparing Bahonar and Shafa hospitals, there were significant differences in the group of reducing or facilitating daily activities, reducing organization costs, ease of use of the system, and user satisfaction (*p* <  = 0.025). There was no significant difference in the comparison between Bahnar and Shahid Beheshti hospitals (*P* >  = 0.144). Comparing Shafa and Shahid Beheshti hospitals, there were significant differences in system security, system ease of use, and user satisfaction (*p* <  = 0.025).

### The IT staff

The usability questions Answered by this group gained a score of 20.375 ± 1.96 out of 30 points. Shahid Beheshti Hospital gained the lowest score (17.5), while Afzalipour Hospital gained the highest (21.75), and the difference between the two was significant (*p* = 0.025). Comparing the feedback of the IT staff of the four hospitals (Afzalipour, Shafa, Bahonar, and Shahid Beheshti) in pairs about the information system of their ESs, all IT staff members believed that there was no significant difference among the hospitals (*p* > 0.05).

**Fig. 2:**
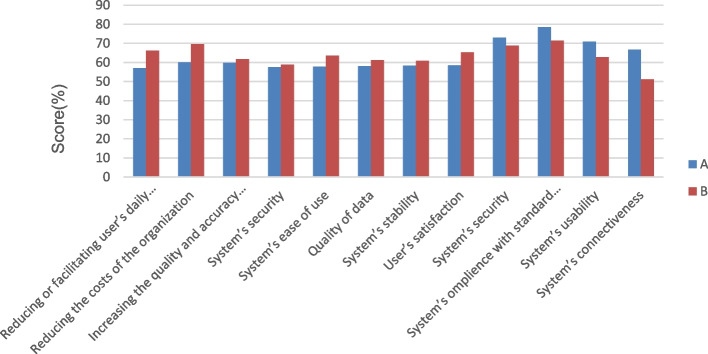
Comparing the usability of the HIS’ presented by Tirajeh Company and Peyvand Dadeha Company. **A** Tirajeh, **B** Peyvand Dadeha

The HIS of three hospitals (Afzalipour, Shafa, and Shahid Beheshti) was designed by Tirajeh, and Peyvand Dadeha Company developed the HIS of Bahonar Hospital. Nurses' opinions on the HIS implemented by Tirajeh and Peyvand Dadeha were collected (Fig. 2). Comparing the HISs designed by the two companies, nurses believed that the two systems were different in their ease of use. Additionally, there was a significant difference in their users’ satisfaction rate (*p* <  = 0.016). For that, a survey compared the nurses' opinions about the emergency module of Tirajeh and Peyvande Dadeh information systems. The comparison showed significant differences in the system's ease of use and user satisfaction (*p* <  = 0.016).

The average usability satisfaction score with the HIS system was 2.93 out of 5. There was no significant difference among the groups regarding the systems offered by the two companies (*P* >  = 0.09). Also, the average usability satisfaction rate of the IT staff with the HIS system was 3.54 out of 5.

## Discussion

In the first phase, by conducting interviews, we identified the usability problems of hospital information systems from the perspective of two groups of users (Emergency department nurses and IT staff). Based on the obtained results, the issues can be divided into four groups: 1) Manual and frequent inserting information in the system and notebooks, 2) Difficulty in searching for a patient, 3) Frequent systems crashing during work, 4) lack of decision support system (DSS). According to the users’ feedback, these problems indicated the poor usability of the system, which increased their working time, reduced their efficiency and led to their dissatisfaction with the system. Rissell et al. also showed that the existence of a long protocol for working with the system and the frequent failure of their system had caused employee dissatisfaction and severe safety issues for patients and employees [45]. Different studies showed that the high usability of a system reduces users’ stress and workload while increasing their satisfaction with the system [45–50].

In the second phase, our results showed significant differences in different hospitals in reducing/facilitating users’ daily activities, reducing organization costs, ease of system usage, and user satisfaction (*p* < 0.03). Because the emergency system in three hospitals was designed by the same company (Tirajeh), but the users’ activities were different, it can be concluded that the difference was due to various needs that the users had in other hospitals. Handayani et al. [48] compared four private hospitals and three public ones. They showed that non-technological factors, such as human factors (e.g., compatibility, self-efficacy) and organizational factors (e.g., management support, user participation), significantly influenced users’ opinions about the ease of use and benefits of HIS.

Our results showed that the opinions of IT staff users in different hospitals were significantly different only about usability (*p* = 0.025). It can be due to using different HISs in other hospitals. However, users of the system designed by Tirajeh were more satisfied with its usability.

Bahonar Hospital had a significant difference from Afzalipur in terms of reducing/facilitating daily activities and organization costs. Bahonar was also significantly different from Shefa Hospital in reducing/promoting daily activities and organization costs, ease of system usage, and user satisfaction. One of the reasons for this difference can be using different HISs used in Bahonar hospital (designed by Linked Data Company) and the ones used in Afzalipur and Shafa hospitals (designed by Tirajeh). Based on users’ feedback, the information system of Bahonar Hospital has more usability than other HISs, which makes its users more satisfied.

According to nurses’ feedback, Shafa and Shahid Beheshti hospitals were notably different regarding ease of system usage and user satisfaction (*p* <  = 0.025). It could be due to the difference in their provided services and workloads. Chou et al. and Chen et al. have also shown that usefulness, ease of use, and attitude toward using positively influence usage, users’ satisfaction, and system acceptance [46, 47]. Due to the reason for having the same HIS in Shafa and Shahid Beheshti hospitals, it can be concluded that the HIS of Shahid Beheshti Hospital has a higher score for ease of system usage and user satisfaction than Afzalipur hospital, and it seems to be more compatible with the hospital’s requirements, which makes it easier to use.

Afzalipur and Shahid Beheshti hospitals had a massive difference in data quality (*p* <  = 0.037). It could be due to the difference in type and form of data storage in their systems. Shahid Beheshti has the highest data quality value (65%), which is acceptable and shows its excellent compatibility with hospital procedures. In a study conducted by Farzdipour et al., the information system was compatible with the work process, which resulted in 86% data quality [50].

Our results showed that users' satisfaction with the study was average and even lower than expected. Users’ dissatisfaction reduces users' interest in using the systems. Subsequently, it lessens their accuracy in using them—this results in increasing users’ fatigue and burnout while decreasing the quality of data.

Managers of healthcare organizations can use the results of our study to tackle the deficiencies of HIS to increase their usability. Our study shows that to enhance the quality of users' interaction with the HIS, it is essential to modify and improve them according to the users’ views and expectations.

### Limitations

Several limitations were identified when conducting our study, including the lack of access to all members of the statistical population during the study (several individuals were excluded from the study as they became infected by COVID-19). Our study was conducted during the COVID-19 pandemic, which made it somewhat difficult and risky to refer and attend hospitals for research. Access was only given to public hospitals, and we could not extend the study to all information systems used in all hospitals of Kerman. Some individuals did not cooperate in data collection and refused to fill out their questionnaires, which was partially resolved with further follow-up.

## Conclusion

The hospital information system is one of the most critical systems used in hospitals and requires more attention in its design and implementation. Failure to solve the problems of hospital information systems and their continuation will have a negative effect on the performance of users and cause them to be dissatisfied with the system. In this study, we identified the usability problems of hospital information systems from the point of view of two groups of users (emergency department nurses and IT staff). Based on the obtained results, from the point of view of the users, the identified problems showed the weak applicability of the system, which has increased the working time, reduced the efficiency, and made them dissatisfied with this system.

Problems and low satisfaction with a system will reduce the desire to use it and minimize accuracy in using the system, which results in increased fatigue, job burnout, and reduced data quality. Managers of healthcare organizations can use the results of this study to demand the removal of these deficiencies to increase the applicability of information systems, and they must modify and improve health information systems according to the views of users and their expectations to improve the quality of users' interaction with the system be enhanced.

## Data Availability

The data generated and analyzed during this study are available from the corresponding author upon reasonable request.

## Abbreviations

HIS: Health Information System
ED: Emergency department
EDIS: Emergency Room Information System
DSS: Decision support system
